# American Association for Labor Legislation

**Published:** 1916-05

**Authors:** John B. Andrews

**Affiliations:** Secretary; 131 E. 23d St., N. Y. City


### CORRESPONDENCE.

This department is intended for the presentation of news and views not coming within the scope of other departments, and particularly to afford opportunity for discussion and criticism of views elsewhere expressed.

### American Association for Labor Legislation.

131 E. 23d St., N. Y. City, Feb. 1, 1916.

Editor Buffalo Medical Journal:—

Dear:—You have doubtless noticed with interest, and possibly with concern, the news in the daily papers concerning the introduction of a bill for compulsory health insurance into the legislatures of New York and Massachusetts.

In brief these bills provide that all manual workers and all those earning less than $100 a month shall be insured, and that the cost of the insurance is to be borne equally by the employer, by the employee, and that the state is to contribute one-fourth of the total. For a total contribution amounting to three per cent of the wages, it is estimated that the insured can be provided with medical care (including medical, surgical, nursing attendance, hospital treatment in necessary cases, and the required medicines and surgical supplies), a cash benefit for a maximum of twenty-six weeks of sickness, and a small funeral benefit for the family, should the wage earner die.

When you go over the sections of the enclosed pamphlet relating to medical care, you will notice the lack of specific detail. This apparent omission arises from the policy of the Social Insurance Committee to postpone drafting these administrative regulations, so closely affecting the medical profession, until the committee could benefit by the advice and co-operation of the profession itself.

This Committee has especially refrained from any attempt to decide what the basis of remuneration, or what the rate of compensation should be, because it considers that these technical questions can best be worked out by physicians themselves. However, the Committee considers that the 100 per cent collections for all insured patients treated and the possibility of putting all practice among wage earners upon a business basis will materially improve the financial status of many practitioners.

The co-operation of the medical men has been particularly welcome. The American Medical Association has been one of the foremost in offering its co-operation, and has appointed a special committee, as you doubtless know, consisting of Dr. Alexander Lambert of New York City, chairman; Dr. Frederic Cotton of Boston; Dr. Henry B. Favill of Chicago. Various local medical bodies have also appointed committees to study the subject, to make recommendations, and to confer with the Social Insurance Committee of the Association for Labor Legislation, New York City.

This committee believes that through cordial co-operation it will be possible to frame regulation which will be satisfactory both to the layman and the practitioner.

Will you not do your part in urging the profession to study the subject and to co-operate?

Very truly yours,
JOHN B. ANDREWS,
Secretary.

